# DNA Damage Response (DDR) and DNA Repair

**DOI:** 10.3390/ijms23137204

**Published:** 2022-06-29

**Authors:** Fiammetta Vernì

**Affiliations:** Department Biology and Biotechnology “Charles Darwin”, Sapienza University of Rome, 00185 Rome, Italy; fiammetta.verni@uniroma1.it

The first aim of cell division is to pass the genetic material, intact and unchanged, to the next generation. This must be achieved despite constant threats by endogenous and environmental agents to the DNA. To counter this hazard, life has evolved several systems to detect DNA damage, signal its presence, and mediate its repair [1,2]. Such responses, which encompass a wide range of cellular events, are biologically significant because they prevent diverse human diseases [3].

It is well known that micronutrients such as minerals and vitamins can produce DNA damage comparable to that induced by carcinogens because of their role as antioxidants and/or cofactors of enzymes involved in DNA metabolism [4]. In this issue, two papers sustain this notion. Gnocchini et al., 2022 [5] provides a further support for the hypothesis that vitamin B6 (pyridoxal 5′-phosphate, PLP) deficiency impairs DNA integrity and can lead to cancer. In this work, the authors show that two PLP inhibitors, 4 deoxypyridoxine and ginkgotoxin, promote loss of heterozygosity (LOH) of the *Drosophila* tumor suppressor *warts (wts)* gene giving rise to the appearance of spectacular tumors on the bodies of adult flies. These tumors, generated by mitotic recombination are, in contrast, rescued by PLP supplementation. LOH is associated with several human tumor suppressors and hence to cancer, thus this finding pinpoints PLP deficiency as a factor which can increase the risk of LOH-associated cancers. Costa et al., 2022 [6] investigate the effects of Zinc in DNA damage response (DDR) modulation. Based on studies revealing that Zn status is significantly compromised in cancer patients, the authors explore the role of Zn in acute myeloid leukemia (AML) cells and compare responses of normal and AML cells to Zn depletion and supplementation. They find that while in normal cells Zn supplementation protects cells from damage accumulation and improves the DDR, in AML cells, Zn potentiates the genotoxicity of DNA-damaging agents by promoting cytotoxic and antiproliferative effects. Underlying mechanisms need to be further understood, however, the Zn dual role in DNA damage modulation leads the authors to hypothesize that Zn might, on one hand, reduce the therapy-related side effects by improving the response of normal cells to the insults of genotoxic therapy and, on the other hand, enhance its effects in malignant cells.

Besides micronutrients, an altered lipid metabolism can also affect genome integrity mainly because of the structural role of lipids as constituents of the nuclear membrane. However, this field is still in its infancy due to the difficulty of applying classic lipid-based techniques to nucleus. The review of Moriel-Carretero 2021 [7] provides a comprehensive insight into the mechanisms behind the role of lipids in genome homeostasis and describes examples of methodological approaches available to study this topic.

DNA double strand breaks (DSBs) represent the most important injury that DNA can experience, thus a full understanding of the mechanisms involved in the repairing of these lesions is instrumental to address potential therapies. The review of De Falco and De Felice, 2021 [8] is mainly focused on non-homologous end joining (NHEJ) and homologous recombination (HR) systems. In addition, the authors analyze DSB repair in Archaea. These organisms represent a good model to study DNA repair because they live in challenging environments characterized by extreme conditions of temperature, salinity, pressure, or pH, that strongly impair DNA integrity. The NHEJ pathway is very rare in Archaea, but in contrast they possess a conserved HR. By considering that the complexity of HR machinery increases with that of the organism, studies in simpler systems, such as Archaea, may be a valid approach to establishing paradigms that can help to understand the more complex human HR pathway. Moreover, studying these organisms could reveal intriguing insights into our own ancestral metabolic history.

Meiotic defects derived from incorrect DNA repair during gametogenesis can lead to mutations, aneuploidies, and generate infertility. Alonso-Ramos et al., 2021 [9], using budding yeast, establish a meiotic role for the conserved Cdc14 phosphatase in the processing and resolution of recombination intermediates. They show that Cdc14 reverses CDK phosphorylation of the Holliday junction resolvase (Yen1) during meiosis until the end of the second division. In addition, they reveal that Cdc14 also promotes Yen1 activity in CDC20-depleted cells, allowing the processing of Holliday junction-containing molecules (JMs) and the formation of crossovers (CO) when other repair pathways are absent.

Cancer is a heterogenous disease involving genetic and environment components that interact with each other. Thus, it is of paramount importance to identify genetic polymorphisms in population that can increase individual susceptibility. The work of Kakhkharova et al., 2022 [10] is focused on the Human NEIL2 DNA glycosylase (hNEIL2) involved in base excision repair (BER), the system that removes oxidative lesions from DNA. The preferred targets of hNEIL2 are lesions in bubbles and other non-canonical DNA structures. In this work, the biochemical characterization of R103W and P304T variants which were taken from databases is described. From the analysis, it emerges that both variants are able to catalyze the base excision and to nick DNA by β-elimination, although with a lower affinity for DNA, compared to the wild-type form. However, the P304T variant displays reduced catalytic activity, while the R103W enzyme is much less affected. Moreover, the P304T variant was also shown to be less proficient than the wild-type in the removal of damaged bases from single-stranded and bubble-containing DNA. Given the importance of oxidative damage, the authors hypothesize that mutations of Pro304 might putatively be associated with cancer, although more evidence is needed.

The goal of cancer therapy is to target tumor cells by exploiting the differences from normal cells to avoid side effects. The review of Huang et al., 2022 [11] shows that the DNA damage response (DDR) is a valuable target for the development of anti-cancer therapies. Although deficiency in DDR genes induces genomic instability and facilitates cancer development, DDR can, conversely, also help cancer cells to resist therapy-induced DNA damage. The authors, after introducing the mechanisms of DDR, discuss how this process can be exploited in cancer therapies based on synthetic lethality and immune checkpoint blockade (ICB). The first strategy exploits the inhibition of the HR system to create an increased sensitivity to PARP inhibitors. The second is based on inhibitors of immune checkpoints proteins that lead cancer cells being attacked by cytotoxic T lymphocytes. Interestingly, tumor-intrinsic DDR features can serve as biomarkers to select suitable patients for immunotherapy. Targeting DDR can also improve cancer immunotherapy by modulating the immune response mediated by cGAS-STING-interferon signaling. The review also includes the contemporary clinical trials of DDR-targeting and ICB therapies in breast, colorectal, and pancreatic cancers.

It is widely accepted that environmental carcinogens can cause cancer and may facilitate progression of the disease. While some of these carcinogens have been identified, other carcinogens present in our environment have yet to be fully defined. Thus, developing strategies to ameliorate genotoxic test is of crucial importance. Kwasniewska and Bara, 2022 [12] performed a comprehensive overview describing the advantages of performing micronucleus (MN) testing in plants to assess the state of the environment. In addition, the authors illustrate the application of molecular cytogenetic techniques to understand the origin, structure, and genetic activity of MN in plants. Moreover, they emphasize the role of the epigenetic modifications in MN formation.

The articles in this special issue add valuable information on different aspects of genome integrity maintenance and provide new avenues for disease prevention and management.
